# Late Injections of Diphtheria Antitoxin

**Published:** 1899-02-25

**Authors:** 


### LATE INJECTIONS OF DIPHTHERIA ANTITOXIN.

So much stress has lately been laid, and rightly laid, upon the importance of using antitoxin in diphtheria at as early a moment as possible, that it is worth while to draw attention to the fact that even after many days one need not hold back from its employment on the idea that it is too late to hope for a good result. A case is reported, by Dr. Daldy,[1] of an infant aged seven months, in which there was a history of croup having been present for seven days. The child was in a most serious condition. The respirations were 36, the pulse 154, and the temperature 99·2. There was croupy cough, great sucking in of ribs and triangles of neck, but no cyanosis. Later plenty of Klebs-Loeffler bacilli were found. Tracheotomy was done, and 1,500 units of antitoxin were injected; it being then the eighth day of the disease. The usual relief to the dyspnœa followed, and the next morning another 1,500 units were injected. The case improved uninterruptedly, and at the time of the report was quite well.

[1] Brit. Med. Jour., Feb. 11.

